# Experimental and Computational Study of Pyrogenic Carbonaceous Matter Facilitated Hydrolysis of 2,4-Dinitroanisole (DNAN)

**DOI:** 10.1021/acs.est.4c01069

**Published:** 2024-05-13

**Authors:** Nourin I. Seenthia, Eric J. Bylaska, Joseph J. Pignatello, Paul G. Tratnyek, Samuel A. Beal, Wenqing Xu

**Affiliations:** † Department of Civil and Environmental Engineering, Villanova University, Villanova, Pennsylvania 19085, United States; ‡ Physical Science Division, Pacific Northwest National Laboratory, Richland, Washington 99352, United States; § Department of Environmental Sciences, The Connecticut Agricultural Experiment Station, 123 Huntington St., New Haven, Connecticut 06511, United States; ∥ OHSU-PSU School of Public Health, Oregon Health & Science University, Portland, Oregon 97239, United States; ⊥ U.S. Army ERDC-CRREL, Hanover, New Hampshire 03755-1290, United States

**Keywords:** 2,4-dinitroanisole (DNAN), pyrogenic carbonaceous matter (PCM), surface-catalyzed hydrolysis, quaternary ammonium (QA) groups, denitration pathway

## Abstract

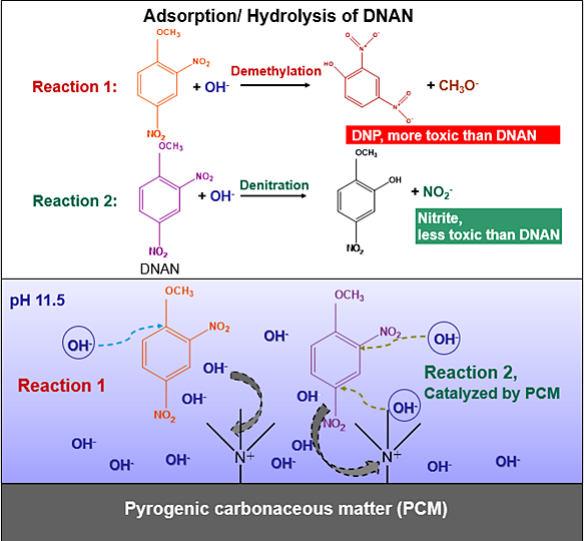

This study investigated the reaction pathway of 2,4-dinitroanisole (DNAN) on the pyrogenic carbonaceous matter (PCM) to assess the scope and mechanism of PCM-facilitated surface hydrolysis. DNAN degradation was observed at pH 11.5 and 25 °C with a model PCM, graphite, whereas no significant decay occurred without graphite. Experiments were performed at pH 11.5 due to the lack of DNAN decay at pH below 11.0, which was consistent with previous studies. Graphite exhibited a 1.78-fold enhancement toward DNAN decay at 65 °C and pH 11.5 relative to homogeneous solution by lowering the activation energy for DNAN hydrolysis by 54.3 ± 3.9%. This is supported by our results from the computational modeling using Car–Parrinello simulations by ab initio molecular dynamics/molecular mechanics (AIMD/MM) and DFT free energy simulations, which suggest that PCM effectively lowered the reaction barriers by approximately 8 kcal mol^–1^ compared to a homogeneous solution. Quaternary ammonium (QA)-modified activated carbon performed the best among several PCMs by reducing DNAN half-life from 185 to 2.5 days at pH 11.5 and 25 °C while maintaining its reactivity over 10 consecutive additions of DNAN. We propose that PCM can affect the thermodynamics and kinetics of hydrolysis reactions by confining the reaction species near PCM surfaces, thus making them less accessible to solvent molecules and creating an environment with a weaker dielectric constant that favors nucleophilic substitution reactions. Nitrite formation during DNAN decay confirmed a denitration pathway, whereas demethylation, the preferred pathway in homogeneous solution, produces 2,4-dinitrophenol (DNP). Denitration catalyzed by PCM is advantageous to demethylation because nitrite is less toxic than DNAN and DNP. These findings provide critical insights for reactive adsorbent design that has broad implications for catalyst design and pollutant abatement.

## Abstract 2

Degradation of 2,4-dinitroanisole can be accelerated by pyrogenic carbonaceous matter, shifting the reaction pathway from demethylation to denitration with the formation of less toxic nitrite.

## Introduction

2,4-Dinitroanisole (DNAN) is one of the most commonly used organic energetic compounds among the insensitive munitions (IM) evaluated by the defense industry as replacements for conventional legacy explosives.^1,2^ Due to its excellent detonation characteristics, low melting point, and lower susceptibility to shock and accidental explosion, DNAN has gained popularity for its use in melt-pour IM formulations.^1−3^ Over 900,000 pounds of DNAN were manufactured in the U.S. in 2019, and production is expected to increase.^4−6^ DNAN may be released from wastewater discharge of formulation manufacturing or munitions load-and-pack (LAP) assembly sites, with reported DNAN concentrations at 128–137 μg L^–1^ from LAP wastewater effluent.^7−10^ Additionally, live-fire training deposits explosives on military training ranges due to incomplete post-detonation.^11,12^ Previous studies found that post-detonation residues of DNAN accounted for 0.006–0.5% of its filler mass in IM projectiles.^11,13^ Due to the relatively low adsorption of DNAN in soil (i.e., *K*_D_ = 0.6–9.1 L kg^–1^), DNAN can readily enter groundwater and undergo long-range transport.^14−16^ Although limited information is available on its human health risks, DNAN is known to be toxic to aquatic organisms, fishes, earthworms, and mammals.^17−19^ Thus, there is an urgent need to develop effective strategies to retain and degrade DNAN and similar compounds present in sources of contamination to prevent their adverse effects.

Among the various treatment technologies, *ex-situ* remediation, such as dredging, raises safety concerns, especially for soils contaminated with explosives at high concentrations.^20,21^ Bioremediation is typically slow and can be ineffective when the munition concentrations are high enough to exert toxicity toward responsible microbial degraders.^22−24^ DNAN has shown toxicity toward earthworms, algae, bacteria, and plants.^25−27^ Previous studies have investigated the use of sulfate green rust, micro or nanoscale zero-valent iron, reactive metals (i.e., nickel), and cathodic processes for reducing energetic compounds in groundwater.^28−31^ However, DNAN reduction generates a mixture of amino products, such as 2,4-diaminoanisole, 2-amino-4-nitroanisole, 4-amino-2-nitroanisole, some of which are hazardous chemicals, and possible human carcinogens.^2,6,31,32^ Oxidation has major drawbacks, as nitroaromatics are difficult to oxidize and thus require strong oxidants (e.g., advanced oxidation and photo-oxidation processes), imposing high operational and maintenance costs.^21,33−38^ Due to the low cost and ease of operation, alkaline hydrolysis with lime addition has been extensively investigated for decontaminating IM residues. In fact, many energetic compounds undergo hydrolysis. For instance, alkaline hydrolysis has proven effective in degrading DNAN and TNT in laboratory studies using IM production wastewater over 8 h but requires a pH of 11.0 or greater.^3,39,40^ Field studies have further demonstrated that TNT had an average half-life of 1.6 days at pH 12.7 in soils treated with 5% by weight of hydrated lime.^41−43^ However, lime treatment has practical limitations as it renders the soil highly alkaline (pH above 11.5). Thus, additional monitoring and evaluation are often required due to concerns over potential pollutants immobilized from the source zone after treatment.^41−44^

Pyrogenic carbonaceous matter (PCM) in the form of activated carbon has been commonly used as a passive adsorbent for immobilizing pollutants in soils and sediments.^45−51^ Recent studies have shown that, in addition to binding pollutants, PCM can promote the degradation of pollutants, including organic pesticides, safeners, and explosives.^52−58^ Of particular interest to this study is that even at neutral pH and room temperature, PCM can promote DNAN hydrolysis with a half-life of 21 d.^59^ This finding has significant implications for *in-situ* remediation. However, reaction pathways and the key properties of PCM responsible for accelerating PCM-facilitated DNAN hydrolysis are unknown. From a practical perspective, it will be interesting to know whether PCM can lower the required pH for effectively accelerating DNAN hydrolysis. Characterization of alkaline hydrolysis products is challenging due to the instability of intermediates and the multiplicity of plausible pathways.^60,61^ While computational studies provided insights into the hydrolysis mechanisms of these compounds,^62−67^ they were focused almost exclusively on the homogeneous solution. To our knowledge, computational studies that explore the role of carbon materials in facilitating contaminant hydrolysis are absent.

The goal of this study is to characterize the reaction pathway for PCM-facilitated DNAN hydrolysis and identify the key properties of PCM responsible for such reactions. Aided by the computational modeling, we evaluated the reaction pathways and provided mechanistic insights into the PCM-facilitated surface hydrolysis. We also assessed the dependence on carbon type using different carbon powders such as graphite, almond shell (AS) char, powdered activated carbon (PAC), and PAC modified with quaternary ammonium (QA) groups and compared their reaction rate constants for DNAN decay. We chose graphite as a model PCM due to its small surface area and minimum surface functionality. We included AS char and PAC as reference materials due to their commercial availability. We further modified PAC with QA groups chemically or physically and investigated their ability to promote DNAN hydrolysis. We hypothesize that incorporating QA groups would introduce positive charges onto the PAC surface, concentrate OH^–^ near its surface, and thus create a local high pH environment to facilitate alkaline hydrolysis of DNAN.^68,69^ Furthermore, we investigated the robustness of the best-performing PCM (i.e., stability and need for regeneration) with ten successive additions of DNAN. The combined approach of experimentation and computational modeling allows us to gain a fundamental understanding of this novel surface process, providing critical knowledge for designing reactive adsorbents to promote the simultaneous adsorption and destruction of nitroaromatic compounds.

## Material and Methods

### Chemicals

Alfa Aesar (Ward Hill, MA): graphite (325 mesh, 99%); sodium phosphate tribasic, anhydrous 100 mesh powder; Sigma-Aldrich (Milwaukee, MI): acetonitrile (HPLC grade, ≥99%); methanol (HPLC grade, ≥99%); glycidyltrimethylammonium chloride (technical, ≥90%); Sigma-Aldrich (St Louis, MO): sodium azide (≥99.5%); 2,4-dinitrophenol (DNP, 5000 μg mL^–1^ in methanol); sulfuric acid (95.0–98.0%); Sigma-Aldrich (Darmstadt, Germany): hydrogen peroxide solution (30% w/w); Fisher Scientific (Pittsburgh, PA): sodium hydroxide (97.9+%), hydrochloric acid (37.1%); Fisher Scientific (Fair Lawn, NJ): sodium bicarbonate (99.7%); ACROS (New Jersey, US): sodium phosphate dibasic, anhydrous; Corigin Solutions, LLC (Merced, CA): almond shell char; Alfa Aesar (Heysham, UK): 2,4-dinitroanisole (DNAN, 98%); powdered activated carbon (PAC; Norit D10). Deionized water (18.2 MΩ cm) was obtained from a Millipore milli-Q-plus water purification system. All chemicals were used as received.

### Preparation of PAC-QA_Phys_

Polydiallyldimethylammonium chloride (polyDADMAC), a QA polymer, was physically adsorbed on PAC following a published procedure with minor modifications.^69^ Briefly, PAC (5 g) was mixed with 16 g of the aqueous polyDADMAC solution (35% by weight) and 16 mL water in a 40 mL polypropylene (PP) centrifuge tube and equilibrated on a tube rotator for 24 h at room temperature to allow polyDADMAC to adsorb. The supernatant was then removed, and the PAC was washed four times each with 30 mL deionized (DI) water to remove non- and loosely bound polyDADMAC and finally dried at 65 °C for 48 h. The obtained modified PAC is abbreviated as PAC-QA_Phys_.

### Preparation of PAC-QA_Chem_

**Scheme 1:**
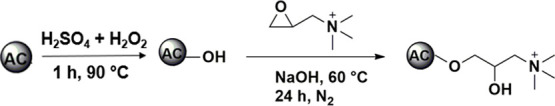
Preparation of PAC-QA_Chem_ from PAC-OH and GTAC

Taking advantage of the reaction between −OH and epoxide,^70^ we reacted PAC with an epoxide derivative (i.e., glycidyltrimethylammonium chloride (GTAC)) to chemically attach QA groups to the surface of PAC (Scheme 1). Briefly, PAC (2 g) was treated with 20 mL piranha solution (a 4.5:1 molar ratio of sulfuric acid and hydrogen peroxide by volume) for 1 h at 90 °C to populate it with −OH groups. The obtained PAC, abbreviated as PAC-OH, was washed with DI water five times and dried in a vacuum oven for 24 h at 60 °C. Subsequently, PAC-OH and GTAC were mixed at three ratios of 1:0.5, 1:2, and 1:4 (by weight) in 15 mL of 0.5 M NaOH solution in a round-bottom flask under nitrogen and stirred for 24 h at 60 °C. After 24 h, the mixture was filtered, and the solid was rinsed with methanol and DI water, followed by Soxhlet extraction with methanol for 48 h to remove loosely bound GTAC. The obtained solid was dried at 60 °C for 24 h. The final product is abbreviated as PAC-QA_Chem_.

### PCM Characterization

The point-of-zero charge of PAC-QA_Chem_ was measured with a Particle Sizer and Zeta Potential Analyzer, Nanobrook Omni (Brookhaven, USA), under the Phase Analysis Light Scattering (PALS) mode with a range of adjusted pH and default parameters at 25 °C in DI water. Zeta potential for three PAC-QA_Chem_ from reagent ratios of 1:4, 1:2, and 1:0.5 were characterized. The point-of-zero charge (PZC) was defined by the point-of-zero proton condition, where the positive charge equals the negative charge. Incorporation of QA groups to PAC was confirmed by X-ray photoelectron spectroscopy (XPS) performed on a PHI 5000 VersaProve with both survey and high-resolutions spectra using a 200 μm, 50 W beam with 117 and 23 eV pass energies, respectively. All XPS data were charge-corrected to adventitious carbon at 284.8 eV binding energy.

The results for the elemental analysis and surface area of graphite, AS char, PAC, PAC-QA_Phys_, and PAC-QA_Chem_ are summarized in Table S1. Elemental analysis of different PCMs was carried out by Galbraith Laboratories (Knoxville, TN) using a Flash 2000 Elemental Analyzer. The surface areas for all PCMs were measured following the Brunauer–Emmett–Teller (BET) method using Micromeritics AutoChem II 2920 with nitrogen.

### Batch Reactor Experiments

DNAN stock solution (10 mM) was prepared by dissolving 39.5 mg DNAN powder in 20 mL of methanol. A small amount of DNAN stock solution (≤0.5% by volume) was introduced into borosilicate glass reactors containing pre-weighed PCM powders, including crystalline graphite powder (10 or 22 g L^–1^), commercially available AS char (10 g L^–1^), PAC-QA_Phys_ (10 g L^–1^) and PAC-QA_Chem_ (10 g L^–1^) to achieve an initial concentration of 50 μM. Thirteen mL of phosphate-carbonate buffer (20 mM, pH 11.5 ± 0.05) with sodium azide (100 mg L^–1^) as an aerobic metabolic inhibitor was introduced to each reactor to reach ∼90% of the total volume. The vials were capped with Teflon-lined septa and placed on an end-to-end rotator at 30 rpm in the absence of light at different temperatures (e.g., 25, 45, and 65 °C). Controls without solids were prepared at the same time. All experiments were conducted at pH 11.5 and in duplicate, unless otherwise indicated. Samples were periodically collected and centrifuged at 4000 rpm for 3 min to separate the aqueous and solid phases. The total mass of DNAN and its transformation products (e.g., DNP and nitrite) were monitored by the sum of the analyte in both the aqueous and solid phases. A 1 mL aliquot of the aqueous phase was withdrawn for chemical analysis. The solid-phase concentrations for DNAN and DNP were calculated using the pre-determined extraction efficiency at pH 3.0 by adding a small amount of HCl to DI water to eliminate the alkaline hydrolysis, following a previously established protocol.^59^ Nitrite extraction efficiency was determined similarly using a mass balance approach, following a previously published method.^71^ The solid-phase extraction efficiencies for DNAN in the presence of various PCMs are summarized in Table S2. For samples containing graphite, 10 mL methanol was added to each reactor after removing the supernatant and then shaken for 3 min, followed by centrifugation for another 3 min at 4000 rpm before chemical analysis of DNAN and DNP. For samples containing AS char, PAC, PAC-QA_Phys_, and PAC-QA_Chem_, the solid phase extraction was performed using accelerated solvent extraction method (Dionex ASE-350, Thermo Fisher Scientific, Waltham, MA) with 50 mL methanol at 100 °C and 1500 psi for 16 min. Afterward, the samples were collected and concentrated from 50 to 10 mL in an evaporator (Genevac EZ-2 Personal Evaporating System, Genevac Ltd., Ipswich, UK) at 25 °C and then passed through a 0.45 μm PTFE syringe filter prior to analysis by high-performance liquid chromatography (HPLC). Nitrite was extracted from the solids with 2 mL of phosphate-carbonate buffer (20 mM, pH 11.5). The supernatants were collected for nitrite analysis using a UV/vis spectrophotometer. To evaluate the stability and reusability of PAC-QA_Chem_, experiments were performed with 10 successive additions of DNAN (50 μM) over 40 days. Both DNAN decay and nitrite formation were monitored. All experiments were conducted in duplicate.

### Adsorption Isotherm

The adsorption isotherm experiments of DNAN on crystalline graphite powder were carried out in duplicate using a constant solid-to-liquid ratio (0.22 g L^–1^) at 25 °C and pH 3.0. DNAN (from 1 to 215 mg L^–1^) was added to batch reactors (each 14 mL) containing pre-weighed graphite, capped, and placed on an end-to-end rotator at 30 rpm in the dark. After 2 days, samples were centrifuged, and the supernatant was analyzed for DNAN by HPLC. The solid-phase concentration of DNAN was calculated using a mass balance approach. The obtained isotherms for DNAN with graphite were fitted with the Langmuir model.

### Analytical Methods

Both aqueous and solid phase extracts of DNAN were analyzed on a Shimadzu HPLC-UV (Model: LC-20ADXR) equipped with a photodiode array (PDA) detector and an XBridge BEH Amide column (2.5 μm, 4.6 mm ID × 100 mm, Waters Corporation, Milford, MA) at 28 °C. The mobile phase was a mixture of acetonitrile and DI water (75:25 by volume) with 2% acetic acid (by volume) at a flow rate of 0.5 mL min^–1^. Both DNAN and DNP were detected at 315 nm, following U.S. EPA method 8330.^72−74^ The calibration curves were established using the purchased analytical standards of DNAN and DNP. DNAN and DNP eluted at a retention time of 6.3 and 5.3 min, respectively, with a signal-to-noise ratio over 40 and a separation factor of >1.4. To evaluate the potential interference from the matrices, we quantified DNP in a mixture of DNAN and DNP. No interference with the matrices was observed. Nitrite was determined colorimetrically by a published method at 543 nm on a UV/vis spectrophotometer (DR6000, HACH, USA).^75,76^ The organic transformation products in the aqueous and solid phase extracts for selected reaction systems were analyzed with an ultra-performance liquid chromatograph interfaced with a high-resolution, quadrupole/time-of-flight mass spectrometer (UPLC-qTOF-MS). Details are provided in Text S1 and Table S3. The data analysis was performed using Origin Lab, and all the reported data were derived from duplicate samples based on the standard error of the regression to determine a 95% confidence interval.

### Computational Modeling

The hydrolysis of solvated 2,4-dinitroanisole (DNAN) was explored using Car–Parrinello simulations.^77−81^ These simulations were performed using a combined ab initio and classical molecular dynamics (AIMD/MM) approach that is part of the pseudopotential plane-wave program (NWPW module) contained in the NWChem quantum chemistry package.^82,83^ This method couples the pseudopotential plane-wave solvers of the Density Functional Theory (DFT) equations^84,85^ for the quantum mechanics region to a molecular mechanics description of a larger region. In the AIMD/MM method, the total energy for the system is given by,
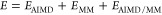
where *E*_AIMD_ and *E*_MM_ are energies for quantum mechanics and molecular mechanics regions, respectively.^86^ Both the PBE96^87^ and PBE0^88^ gradient-corrected exchange-correlation functionals were used. The PBE0 hybrid functional was solved using the technique developed by a previously published method.^77,82,89,90^ The valence electron interactions with the atomic H, C, N, and O cores were approximated using a generalized norm-conserving Hamann pseudopotentials^91,92^ modified into a separable form as suggested by Kleinman and Bylander.^93^ The original pseudopotential parametrizations suggested by Hamann were too “hard”, and softer pseudopotentials were constructed by increasing the core radii, H: rcs = 0.8 au, rcp = 0.8 au; C: rcs = 0.8 au, rcp = 0.85 au, rcd = 0.85 au; N: rcs = 0.7 au, rcp = 0.7 au, rcd = 0.7 au; O: rcs = 0.7 au, rcp = 0.7 au, rcd = 0.7 au. The electronic wave functions were expanded using a plane-wave basis set with periodic boundary conditions, sampled at the Γ point, with a wave function cutoff energy of 100 Ry and a density cutoff energy of 200 Ry. The Car–Parrinello equations of motion were integrated in the presence of Nose-Hoover thermostats,^94−96^ coupled to the electronic and ionic degrees of freedom, at *T* = 300 K with a time step of 0.17 fs and a fictitious mass of 750 au. The SPC/E MM potential for water was used in this study.^97^ The AIMD/MM free energy simulations used in this work followed our previous study.^66^

## Result and Discussion

### Effect of Graphite Powder on DNAN Degradation

**Figure 1:**
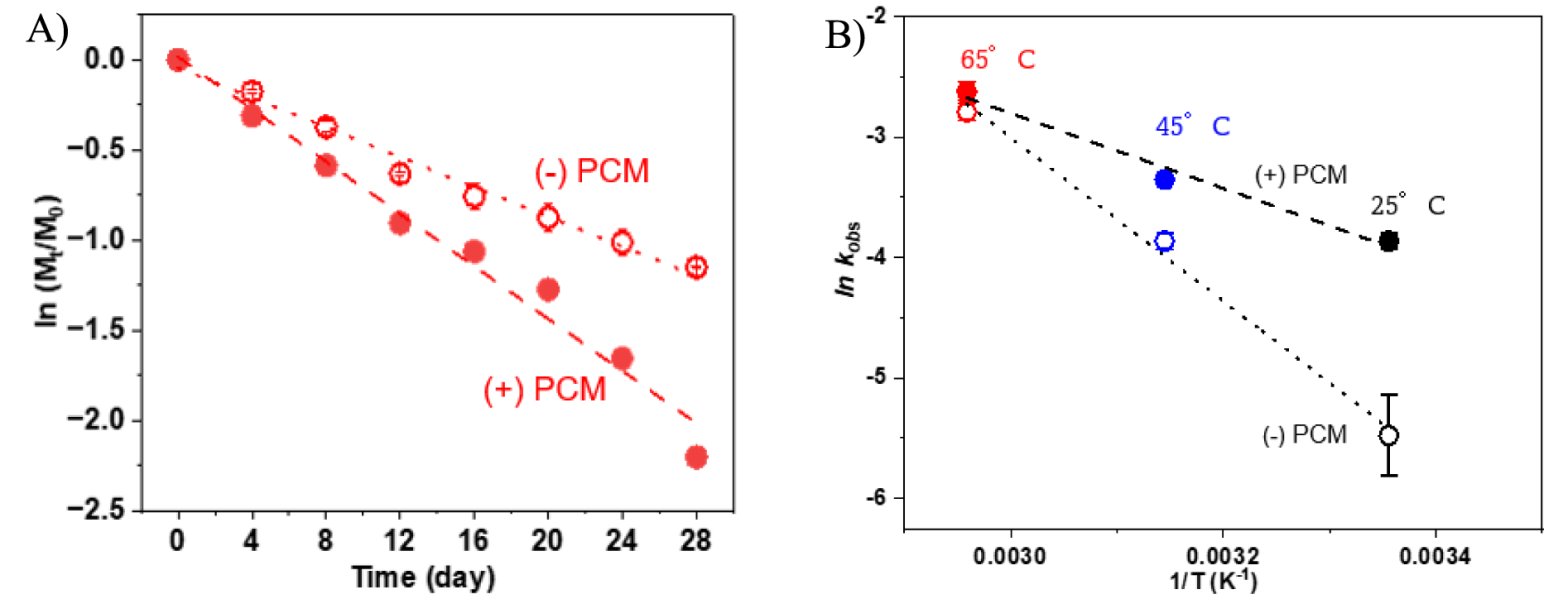
(A) DNAN degradation in the presence and absence (solid circle, empty circle) of 22 g L^–1^ graphite for 28 days at pH 11.5 at 65 °C. (B) Linearized Arrhenius plot for DNAN decay in the presence and absence of graphite at 25 °C (solid circle, empty circle), 45 °C (solid circle, empty circle) and 65 °C (solid circle, empty circle), at pH 11.5. Solid circles represent DNAN decay in the presence of graphite at 25, 45, and 65 °C [ln *k*_obs_ = (−3113.8 ± 432.5) (1/*T*) + (6.5 ± 1.4); *R*^2^ = 0.98] and empty circles represent DNAN decay in the absence of graphite 25, 45, and 65 °C [ln *k*_obs_ = (−6781.7 ± 538.4)(1/*T*) + (17.3 ± 1.7); *R*^2^ = 0.99]. The initial DNAN concentration was 50 μM. The reported data were derived from duplicate samples based on the standard error of the regression to determine a 95% confidence interval.

As shown in Figure 1A, DNAN decay followed pseudo first-order kinetics with or without graphite, a model PCM, at pH 11.5 and 65 °C. The observed rate constants (*k*_obs_) are 0.073 ± 0.004 d^–1^ with graphite and 0.041 ± 0.002 d^–1^ without graphite, corresponding to the half-lives (*t*_1/2_) of 9.5 ± 0.5 days and 17.0 ± 0.9 days, respectively. The addition of graphite clearly accelerated the degradation of DNAN with a 1.78-fold enhancement at pH 11.5 and 65 °C. Rate enhancement by graphite was observed at other temperatures as well (i.e., 25 and 45 °C). For example, in the presence of graphite, the *k*_obs_ increased from 0.021 ± 0.001 d^–1^ at 25 °C to 0.035 ± 0.001 d^–1^ at 45 °C, corresponding to the *t*_1/2_ of 33.1 ± 1.6 days and 18.3 ± 0.5 days, respectively (Figure S1). In the absence of graphite, the calculated *k*_obs_ for DNAN are 0.004 ± 0.001 d^–1^ and 0.022 ± 0.002 d^–1^, respectively (Figure S1). All values for *k*_obs_ and *t*_1/2_ with or without graphite are summarized in Table S4. The calculated extraction efficiencies for DNAN from graphite were 82.0 ± 0.6 and 82.1 ± 0.1% (Table S2) over 24 and 48 h, respectively. As shown in the adsorption isotherm (Figure S2), the adsorbed DNAN concentration increased as the concentration of DNAN increased in solution, which later plateaued off. The adsorption isotherm was fitted with the Langmuir model (*R*^2^ > 0.99). The maximum adsorption capacity (*Q*_max_) based on the Langmuir model was 3.57 mg g^–1^. For an initial DNAN concentration of 50 μM, the percentage DNAN loss from the aqueous phase due to adsorption was 91.03 ± 0.2% after 2 days at pH 3.0 and 25 °C. The activation energy (*E*_a_) was calculated by the Arrhenius equation (*k*_obs_ = Ae^–*E*a/*RT*^), where *k*_obs_ is the observed rate constant, *A* is the exponential factor, *E*_a_ is the activation energy (J mol^–1^), *R* is the gas constant, and *T* is the temperature (K). The *E*_a_ for DNAN hydrolysis in the presence and absence of graphite are 25.9 ± 3.6 and 56.4 ± 4.5 kJ mol^–1^, respectively (Figure 1B). Thus, graphite under these conditions (22 g L^–1^ graphite, pH 11.5) lowers the *E*_a_ for DNAN hydrolysis by 54.3 ± 3.9%.

**Figure 2:**
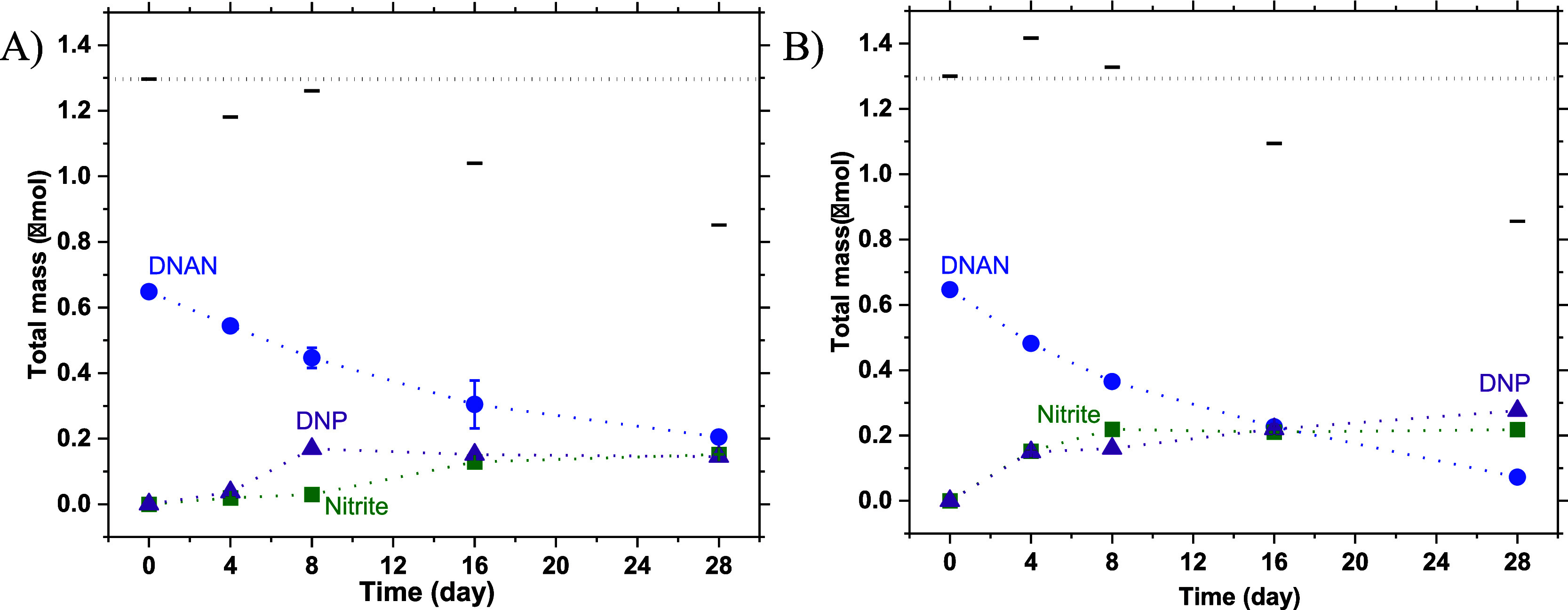
Formation of nitrite (square) and DNP (triangle) during the degradation of DNAN (circle) in the (A) absence and (B) presence of 22 g L^–1^ crystalline graphite powder at 65 °C for 28 days at pH 11.5. The initial concentration of DNAN was 50 μM. The mass balance (---) on the total nitrogen was calculated as 2M_DNAN_ + 2M_DNP_ + M_nitrite_, in μmol, whereas the dotted line (······) denotes the initial mass of the total nitrogen. The reported data were derived from duplicate samples.

**Figure 3:**
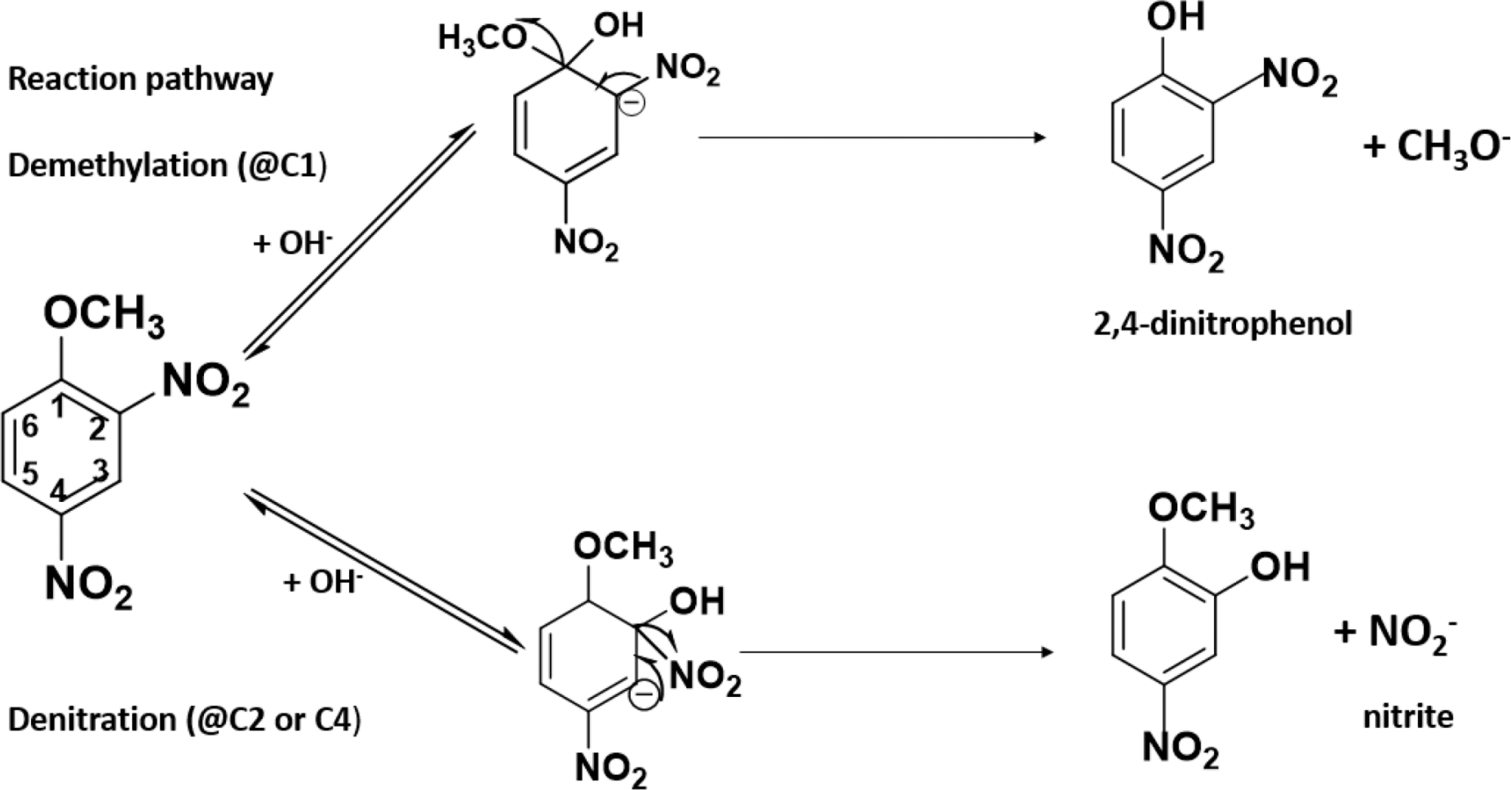
Two hydrolysis pathways of DNAN; demethylation (above): OH^–^ attacks at the C1 position of DNAN and produces a methoxide leaving group and 2,4-dinitrophenol (DNP), and denitration (below): OH^–^ attacks at the C2 or C4 position of DNAN and produces nitrite.

The transformation products of DNAN include nitrite, determined colorimetrically, and DNP, determined by UPLC-qTOF-MS (*m*/*z* = 183). The extraction efficiencies for DNP and nitrite on graphite were 82.1 ± 0.3 and 98.5 ± 0.2%, respectively. Their masses (in μmoles total in the aqueous plus solid phases) in the absence or presence of graphite over a 28-day reaction period at 65 °C and at pH 11.5 are plotted in Figure 2. Also plotted in Figure 2 are the total quantified nitrogen (in μmoles), defined as the sum of the mass of nitrite, twice the mass of unreacted DNAN, and twice the mass of DNP. A range of 75.2–110.0% of the total nitrogen was recovered for the transformation of DNAN with graphite over the experimental time frame. The missing mass of nitrogen could be due to the formation of intermediate products that were not stable and were difficult to detect. As shown in Figures S3 and S4, a new peak appeared with a considerably shorter retention time than DNAN (5.82 min), suggesting that the product is less hydrophobic and more polar. Our results suggest that DNP is the major organic product of DNAN in reactions with and without graphite. The yield of nitrite, defined as the molar ratio of [nitrite formed]/[DNAN decayed] × 100%, over 28 days, was 50.0% or 33.3% with or without graphite, respectively. Similarly, the yield of DNP, defined as the molar ratio of [DNP formed]/[DNAN decayed] × 100%, over 28 days was 39.7% or 33.3% with or without graphite, respectively. Overall, our results suggest that graphite accelerated DNAN hydrolysis favoring denitration over demethylation pathways (Figure 3), and thus affected the product distribution, especially for nitrite. Moreover, no nitrite was formed at pH 11.5, 12.0, or 13.0 at 25 °C. Although no significant DNAN decay was observed at pH 11.5 and 25 °C, we found a 1:1 molar ratio yield of DNP from DNAN decay at higher pH (i.e., 12.0 and 13.0) at 25 °C over 8 days (Figure S5). The observation of a 1:1 conversion between DNAN and DNP was consistent with previous studies at pH 12.0 and 13.0 in the absence of PCM. We hypothesize that an elevated temperature may shift the reaction toward the denitration pathway and thus favor the nitrite formation during DNAN alkaline hydrolysis in the absence of PCM.

Compared to previous studies^59−61,98^ where DNAN hydrolysis was monitored in a different buffer (phosphate), our observed reaction rate for DNAN hydrolysis was slower. To investigate the possible catalytic effects of phosphate buffer toward DNAN hydrolysis, DNAN decay was monitored at pH 11.5 in phosphate buffer (20 mM) at 25 and 65 °C. As shown in Figure S6, The obtained rate constants (*k*_obs_) were 0.029 ± 0.001 d^–1^ and 0.012 ± 0.006 d^–1^ for reaction systems with and without graphite at pH 11.5 and 25 °C. Faster DNAN decay was observed at pH 11.5 and 65 °C, with *k*_obs_ of 0.075 ± 0.004 d^–1^ and 0.047 ± 0.002 d^–1^ for systems with and without graphite (Table S4). Compared to the rate constants in the phosphate-carbonate system (20 mM, pH 11.5), phosphate buffer enhanced the DNAN decay by up to 3 times. The yield of nitrite was 1.2% or 65% without or with graphite, respectively, and the yield of DNP was 80% or 30% without or with graphite, respectively, over 5 days (Figure S7). No nitrite was detected in solutions containing only DNAN, DNP, or a mixture of DNAN and DNP in phosphate-carbonate buffer at pH 11.5, confirming that there was no interference in our reaction system for nitrite quantification.

### Computational Modeling

Previous modeling efforts identified two hydrolysis pathways of DNAN, both involving nucleophilic aromatic substitution by OH^–^ (Figure 3). In the first reaction pathway, an attack of OH^–^ at the C1 position of DNAN produces a methoxide leaving group and DNP, which have been reported by many studies of DNAN hydrolysis in solution.^60,61,66,99−103^ This is problematic because DNP is more toxic than DNAN.^17^ In the second pathway, DNAN undergoes direct substitution (Sub@2 or Sub@4) by the attack of OH^–^ and releases nitrite.^66^ However, even though the second pathway is more thermodynamically favorable, it seems to be kinetically limited since it has not been observed in any experimental studies of DNAN hydrolysis in aqueous solution.^60,66^ The present study demonstrates that elevated temperature favors the denitration pathway, especially in the presence of graphite.

To provide atomistic insights into the observed phenomenon, we investigated the mechanism of DNAN hydrolysis and its different pathways using ab initio molecular dynamics/molecular mechanics (AIMD/MM) and DFT free energy simulations. Our calculations show that the energy barriers associated with the nucleophilic aromatic substitution pathways (i.e., denitration), yielding a less toxic nitroanisole and nitrite, are quite high (Δ*G*^‡^ > 29 kcal mol^–1^ PBE0 AIMD/MM). For DNAN, the most favorable pathway was predicted to follow a Muro–Tomilla reaction mechanism, which is a three-step process. Initially, hydroxyl attacks the C1 carbon, forming a Meisenheimer complex at the C1 arene carbon, represented as C1–(OCH_3_)OH^–^. In the next step, the methoxy anion (−OCH_3_) at the C1 arene carbon dissociates, which has the highest energy barrier in the overall pathway. The reaction then proceeds with the transfer of a proton from the hydroxyl group (C1–OH), acting as an acid, to the dissociated methoxy group, serving as a base. This proton transfer leads to the creation of methanol (CH_3_OH) and an aryloxy anion (2,4-dinitrophenoxide). Methanol forms as the methoxy group acquires a proton, and the aryloxy anion emerges from the loss of a proton from the hydroxyl group attached to the aromatic ring, leaving a negatively charged oxygen atom behind. Moreover, upon the introduction of a low pH buffer, the aryloxy anion readily converts into DNP. However, compared with the Muro-Tomilla reaction mechanism, denitration is preferable because DNP as a product is even more toxic than DNAN.

Our previous work suggests that solvent properties are critical in influencing reaction dynamics in solution, particularly in specialized environments like nano-pores.^100^ For instance, solvents with a lower dielectric constant can significantly impact the thermodynamics and kinetics of hydrolysis reactions. As the dielectric constant lowers in solvents, the reaction energy decreases due to less hydration energy. This effect is especially evident in nucleophilic aromatic substitution pathways, where the solvation energy of the hydroxide ion plays a dominant role. The less solvated, the more nucleophilic the hydroxide ion is. The physics and chemistry behind this analogy are based on the fact that confinement of the reaction species in nano-pores makes it less accessible to solvent molecules, resulting in a weaker dielectric constant (i.e., <78). To test this hypothesis and provide a more quantitative analysis, we performed AIMD/MM free energy simulations using expanded slabs and unit cells, focusing on the interaction of DNAN, hydroxide ions, Na^+^, and multiple water molecules sandwiched between two graphene layers.

**Figure 4:**
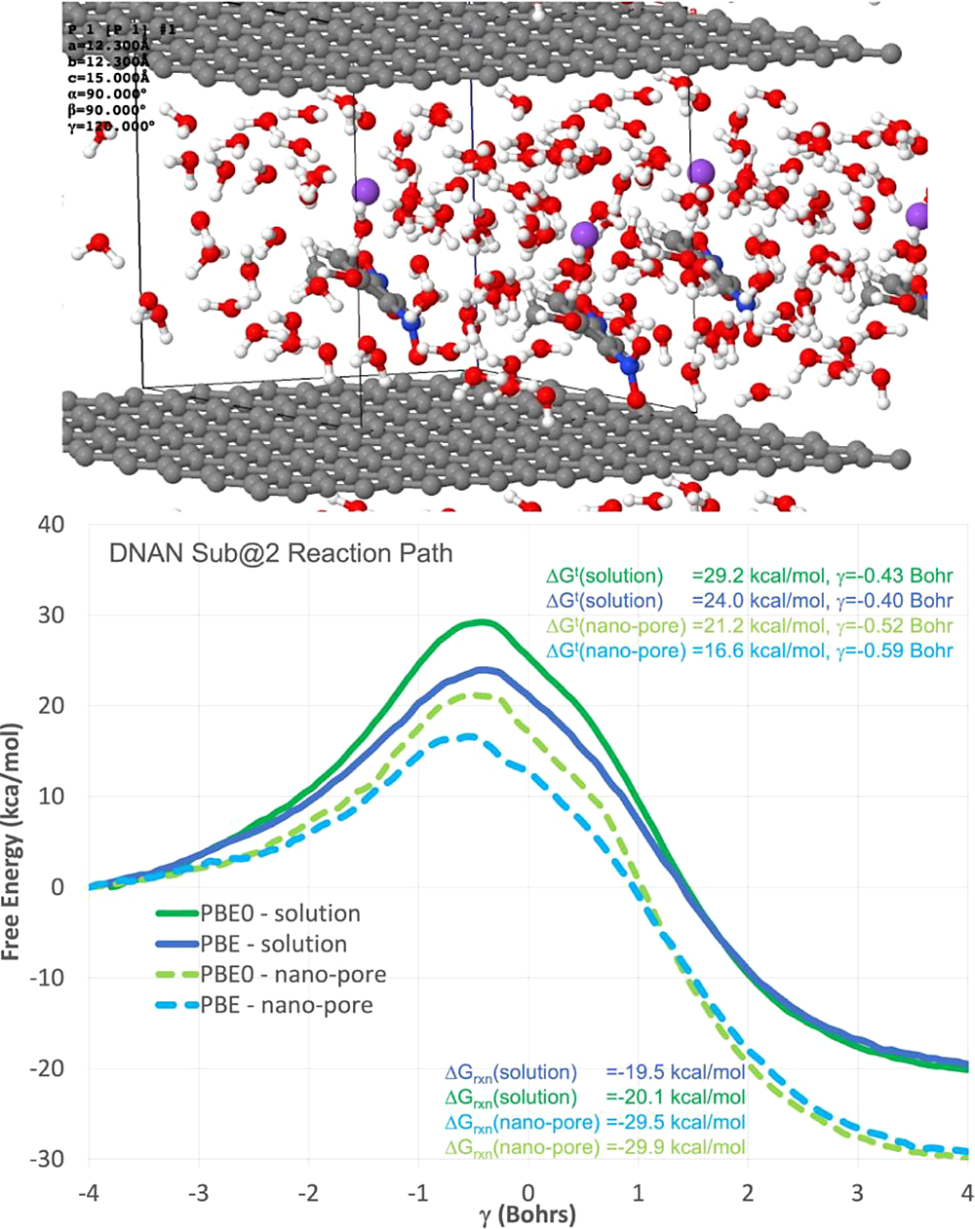
(Top) Molecular snapshot from an AIMD/MM simulation: DNAN + [OH−] → DNAN-2-OH + nitrite in a nano-pore, containing DNAN, hydroxide, Na^+^ counterion, and 43 H_2_O, at a concentration of 1.3 M. (Bottom) Reaction pathways: Nucleophilic aromatic reaction of DNAN + [OH−] → DNAN-2-OH + Nitrite in solution and within a nano-pore, investigated using PBE and PBE0 AIMD/MM free energy simulations with WHAM. Each pathway used approximately 0.5 ns. of simulation time.

As shown in Figure 4, we presented computational results of DNAN hydrolysis pathways, with a focus on reaction nano-porous environments. The upper panel of the figure provides a molecular snapshot from the AIMD/MM simulation, showcasing the intermediate stage of DNAN reacting with a hydroxide ion within a nano-pore structure. This visualization aids in understanding the spatial arrangement of molecules and the context of the reaction within the confined space.

The lower panel of Figure 4 warrants a more detailed discussion for clarity. It depicts the reaction energy profiles for the hydrolysis of DNAN, both in bulk aqueous solution and within the nano-pore environment. The *x*-axis represents the reaction coordinate, which is a schematic representation of the progression from reactants to products through various transition states and intermediates. The *y*-axis corresponds to the Gibbs free energy changes (Δ*G*), providing insights into the thermodynamic favorability of each step in the pathway.

To interpret this panel, one should note the relative heights of the energy barriers (peaks) and the energy of the reactants and products (valleys). Lower barriers correspond to more kinetically accessible reactions. In the nano-pore environment, as shown by the data, the energy barriers are significantly reduced, suggesting a catalytic effect due to confinement. This reduction is quantified by a decrease in Δ*G* of approximately 8 kcal mol^–1^ compared to the bulk solution, indicating that the reactions are not only more thermodynamically favorable but also kinetically accelerated in the nano-pore.

Furthermore, we illustrated two distinct pathways of hydrolysis, denoted as PBE and PBE0, based on the exchange-correlation functionals used in our simulations. The pathways are marked by different colors, with the corresponding energy barriers labeled. We used the weighted histogram analysis method (WHAM)^104,105^ to estimate these free energy barriers from our AIMD/MM simulations. WHAM combines data from multiple simulations that are windows of the reaction coordinate to calculate a detailed energy landscape, enabling us to identify the energetic barriers critical for understanding reaction kinetics. This method is particularly effective for revealing how nano-pore environments influence the hydrolysis of DNAN.

In conclusion, the results encapsulated in Figure 4 demonstrate that nano-pore environments can significantly alter the hydrolysis mechanism of DNAN, leading to potentially less toxic products. This has profound implications for understanding the environmental fate of DNAN and designing remediation strategies for nitroaromatic compounds.

### Dependence on the PCM Type

The decay of DNAN was monitored in the presence of graphite, AS char, PAC, PAC-QA_Phys_, or PAC-QA_Chem_ at pH 11.5 and 25 °C. The incorporation of QA groups onto PAC was confirmed by XPS and zeta potential measurements. Specifically, the total nitrogen content (Figure S8) at 402.84 eV increased from 0% to 0.74%, 0.93%, and 1.43% for three PAC-QA_Chem_ prepared at reagent ratios of 1:0.5, 1:2, and 1:4 (i.e., PAC-OH vs GTAC) and PAC-QA_Phys_. No nitrogen was found in the unmodified PAC. The zeta potential of all three PAC-QA_Chem_ decreased as the pH increased from 2.0 to 10.0 (Figure S9), whereas the PZC values increased from 4.0, 4.3, to 4.5 as the N content increased. Except at pH 6.0, all QA-modified carbons had greater (less negative) zeta potential than the unmodified PAC. Only PAC-QA_Chem_ prepared at 1:4 PAC-OH to GTAC ratio (by weight) was used for the subsequent experiments due to its highest N content and PZC.

**Figure 5:**
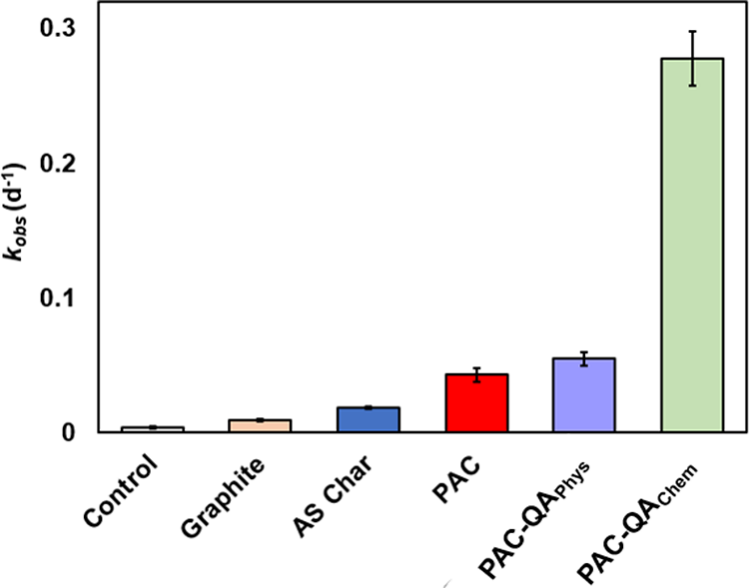
Comparison among the observed reaction rate constants of DNAN in the presence of different PCM types. Reaction conditions: [DNAN]_0_ = 50 μM, solid-to-liquid ratio = 10 g L^–1^, *T* = 25 °C, pH= 11.5 (20 mM phosphate-carbonate buffer). The reported error bars were derived from duplicate samples.

As shown in Figures S10 and S11, pseudo first-order DNAN decay kinetics were observed in the presence of all PCM types. Overall, the presence of PCM, regardless of the carbon types, promoted DNAN degradation at pH 11.5 and 25 °C, whereas no significant DNAN degradation was observed in a homogeneous solution. The *k*_obs_ values of DNAN hydrolysis in the presence of PCM are compared across different types (Figure 5), and the associated *k*_obs_ and half-lives (*t*_1/2_) for DNAN in the presence of graphite, AS char, PAC, PAC-QA_Phys_, and PAC-QA_Chem_ are summarized in Table S5**.** Among the PCMs tested, PAC-QA_Chem_ performed the best, with a *k*_obs_ value of 0.278 ± 0.034 d^–1^, corresponding to a *t*_1/2_ of 2.5 ± 0.3 days at pH 11.5 and 25 °C (Figure 5). This corresponds to a 70-fold enhancement compared to DNAN hydrolysis in a homogeneous solution. Enhancement factors for other PCM relative to the homogeneous solution under the same condition are 2.4 (graphite), 4.6 (AS char), 10.8 (PAC), and 13.8 (PAC-QA_Phys_). The extraction efficiencies of DNAN on graphite and PAC-QA_Chem_ were above 80% (Table S2). However, the extraction efficiencies of DNAN from AS char, PAC, and PAC-QA_Phys_ were lower (i.e., 56.2 ± 0.1, 21.3 ± 3.4, and 31.1 ± 1.2%, respectively), which could be attributed to the surfaces of char and PAC are known to bind organic compounds irreversibly.^106−108^ To validate our results, we performed the solid-phase extraction for two separate time points (i.e., 24 and 48 h). No significant differences in the obtained extraction efficiencies were observed (Table S2). When *k*_obs_ was normalized by the PCM dosage (10 g L^–1^) (Figure S12A), DNAN decay in the presence of PAC-QA_Chem_ was still 29.3 and 6.5 times higher than that of graphite and PAC, and >90% of DNAN was on the solid phase. Similarly, when *k*_obs_ was normalized by both PCM dosage (10 g L^–1^) and surface area (Figure S12B), DNAN decay in the presence of PAC-QA_Chem_ was still 5.6 times higher than that of graphite. These results suggest that in addition to the PCM dose and surface area, other factors such as the surface functional group identity may be important for PCM-facilitated DNAN hydrolysis. Specifically, we postulate that the density of QA on PAC can increase OH^–^ concentration near the surface (Figure S13) and thus contribute to the observed high reactivity toward DNAN decay, which is supported by previous studies.^69,109^ Moreover, PCM as an apolar surface, can concentrate both DNAN and OH^–^ on the surface, where rate acceleration is due at least in part to the high effective concentrations of the reactants on the surface, thus facilitating alkaline hydrolysis.^71^ The differences between PAC-QA_Chem_ and PAC-QA_Phys_ can potentially be explained by the adsorption of polyDADMAC, which may take up adsorption sites that are also reactive for DNAN decay.

### Stability and Reusability of Chemically Modified PAC-QA

**Figure 6:**
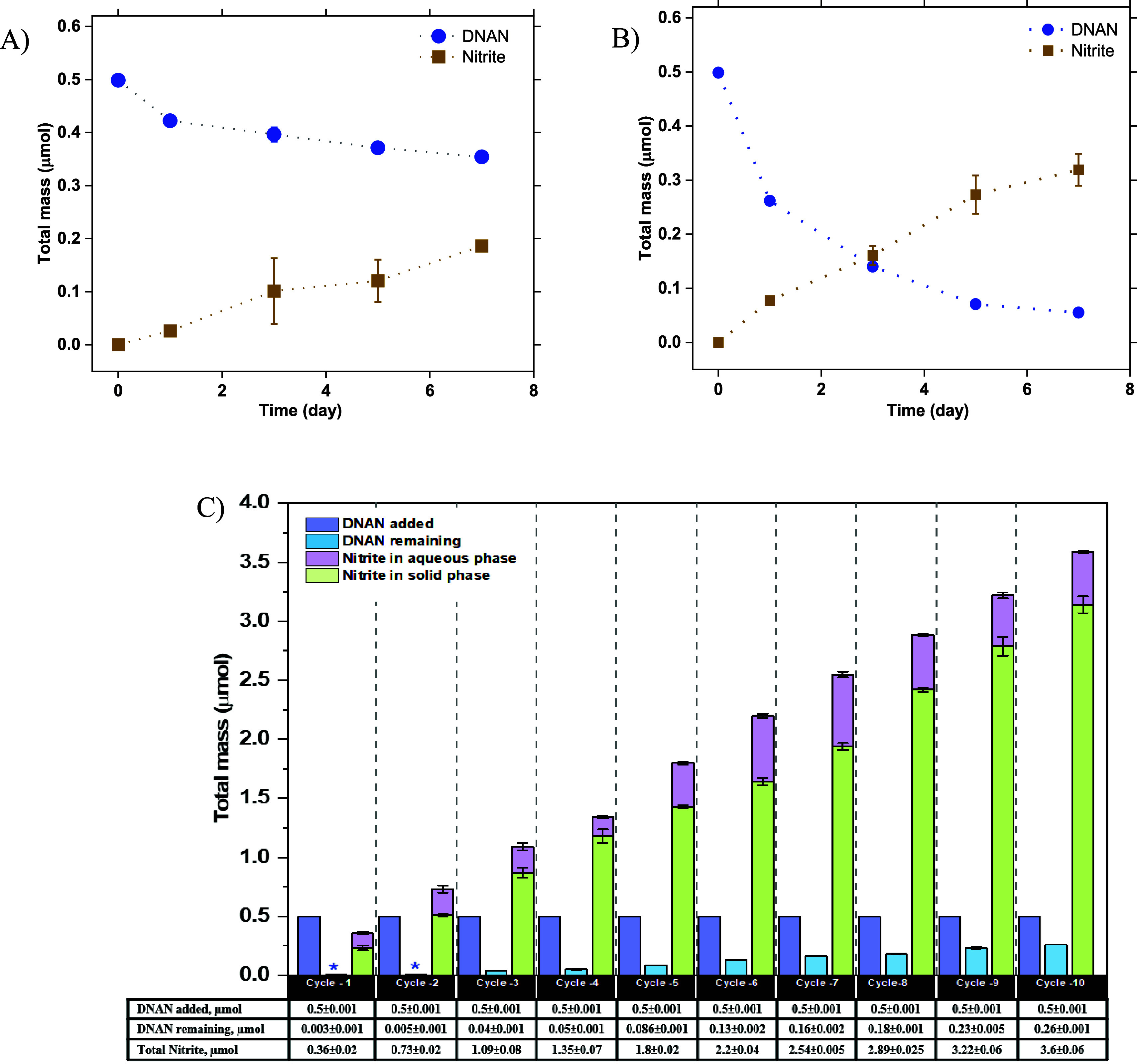
Formation of nitrite (square) during the degradation of DNAN (circle) in the presence of (A) PAC and (B) chemically grafted PAC-QA_Chem_ at pH 11.5 for 7 days. Uniform conditions: [DNAN]_0_ = 50 μM, [PAC] and [PAC-QA] = 10 g L^–1^, *T* = 25 °C, pH = 11.5 (20 mM phosphate-carbonate buffer); controls without PCM were simultaneously performed at pH 11.5. (C) DNAN hydrolysis and nitrite formation with and without chemically grafted PAC-QA_Chem_ over 40 days with ten successive additions of 0.5 μmol DNAN to the system. The remaining DNAN and nitrite formation were measured after every 4 days (i.e., cycles 1–10). Blue asterisk (*) represents values that are far below the present scale. Uniform conditions: [DNAN]_0_ = 0.5 μmol, [PAC-QA_Chem_] = 10 g L^–1^, *T* = 65 °C, pH = 11.5 (20 mM phosphate-carbonate buffer); controls without PCM were simultaneously performed at pH 11.5. The reported error bars were derived from duplicate samples.

Earlier in Figure 2, we observed nitrite formation during DNAN hydrolysis with graphite, and this knowledge can be applied to other PCMs, too. As shown in Figure 6A,B, PAC-QA_Chem_ hydrolyzes DNAN at pH 11.5 and 25 °C more rapidly than unmodified PAC, where the nitrite yields were 125.1 ± 0.3 and 72.0 ± 0.01%, respectively. The extraction efficiencies of nitrite from PAC and PAC-QA_Chem_ were 99.1 ± 0.3 and 99.0 ± 0.2%, respectively. The formation of nitrite in the presence of PAC and PAC-QA_Chem_ confirms other PCMs, in addition to graphite, can also accelerate DNAN hydrolysis at pH 11.5, favoring denitration over demethylation, even at 25 °C. To investigate the stability and reusability of PAC-QA_Chem_, DNAN hydrolysis and nitrite formation with and without PAC-QA_Chem_ were monitored at pH 11.5 and 65 °C with 10 successive additions of DNAN over 40 days (Figure 6C). DNAN to PAC-QA_Chem_ ratio of 5 μmol g^–1^ was chosen using the solubility of DNAN in water (0.213 ± 0.012 g L^–1^, at 25 °C) as a constraint.^2,75,110^ The experiments were carried out at 65 °C to speed up the reaction rate and make a shorter evaluation time possible. Both nitrite formation and DNAN decay were observed at a nitrite/DNAN molar ratio of approximately 0.72:1 for all cycles (Figure 6C), which is consistent with the result obtained for DNAN hydrolysis with PAC-QA_Chem_ at 25 °C (Figure 6B). The molar mass of remaining DNAN and formed nitrite in the solid phase increased over ten consecutive cycles, whereas a negligible amount of DNAN was found in the aqueous phase. Overall, an average of 94% of total DNAN was transformed over the ten cycles, highlighting the robustness of the modified PCM. By contrast, no significant decay of DNAN or formation of nitrite was observed in the carbon-free controls under the same conditions. Our results suggest that the reactivity of PCM can be significantly enhanced by chemically grafting QA groups onto PCM, and the reactivity of the modified PCM can be maintained for at least ten cycles.

### Environmental Significance

Hydrolysis of nitroaromatic compounds, such as DNAN and TNT, has been extensively investigated as a cleanup method for wastewater, contaminated groundwater, and soils. However, the formation of phenolic products from hydrolysis in bulk solution has raised concerns due to their higher toxicity than parent compounds and their corrosiveness.^111^ In this study, we demonstrated that PCM can significantly accelerate the rate of DNAN hydrolysis and thus reduce the required pH for effective DNAN hydrolysis. Moreover, we showed that PCM favored the denitration reaction pathway over demethylation. As a result, the product distribution was shifted from DNP to nitrite and 2-methoxy-4-nitrophenol, whereas both of them are less toxic than DNP and the parent compound (i.e., DNAN). 2-methoxy-4-nitrophenol is an irritant (skin, eye, respiratory), whereas DNP has acute toxicity and is considered a health and environmental hazard.^112,113^ Furthermore, our results from the computational modeling suggest that PCM effectively lowered the reaction barriers by approximately 8 kcal mol^–1^ compared to the bulk solution. We propose that PCM can affect the thermodynamics and kinetics of hydrolysis reactions by confining the reaction species near PCM surfaces, thus making them less accessible to solvent molecules and creating an environment with a weaker dielectric constant that favors nucleophilic substitution reactions.

In addition, we successfully prepared PCM-QA_Chem_ by chemically grafting QA groups on the PCM surface. The obtained PCM-QA_Chem_ showed the highest reactivity in catalyzing DNAN hydrolysis, more than 70 times faster than the DNAN hydrolysis in the bulk solution. Furthermore, no significant loss of reactivity of PCM-QA_Chem_ was observed over 10 consecutive additions of DNAN near its water solubility limit. Overall, this study provides critical insights that elucidate the reaction mechanisms for PCM-facilitated surface hydrolysis and identifies critical properties of PCM that are responsible. These findings may lay the groundwork for the reactive adsorbent design that can be implemented for both *in-situ* and *ex-situ* processes to enhance surface hydrolysis reactions of contaminant degradation, where PCM can be utilized not only to concentrate but also destroy contaminants on the surface and, thus, eliminating the need for material regeneration. Further research is needed to understand the scale-up feasibility, life-cycle analysis, and life-cost analysis of the proposed technology.

## Supporting Information Available

Details of the UPLC-qTOF-MS analysis; elemental analysis of different PCM types and extraction efficiency of DNAN; fitting of the rate constants of the DNAN degradation; adsorption isotherm of DNAN on graphite; quantification of the DNAN transformation products; XPS survey spectra and zeta potential for several PCM types; and comparison among different PCMs by normalized rate constants (PDF)
