# Supplementary material for: Experimental and Computational Study of Pyrogenic Carbonaceous Matter Facilitated Hydrolysis of 2,4-Dinitroanisole (DNAN)
Source: Environ Sci Technol. 2024 May 13;58(21):9404–15. doi: 10.1021/acs.est.4c01069 (PMC11137867; doi:10.1021/acs.est.4c01069)
Supplement: Supplementary file 1 — es4c01069_si_001.pdf [file es4c01069_si_001.pdf]

SUPPORTING INFORMATION

Experimental and Computational Study of Pyrogenic Carbonaceous Matter Facilitated Hydrolysis of 2,4-Dinitroanisole (DNAN)

Nourin I. Seenthia[1], Eric J. Bylaska[2], Joseph J. Pignatello[3], Paul G. Tratnyek[4], Samuel A. Beal[5], Wenqing Xu[1,*]

[1]Department of Civil and Environmental Engineering, Villanova University, Villanova, Pennsylvania 19085, United States

[2]Physical Science Division, Pacific Northwest National Laboratory, Richland, WA 99352, USA

[3]Department of Environmental Sciences, The Connecticut Agricultural Experiment Station, 123 Huntington St., New Haven, CT 06511, USA

[4]OHSU-PSU School of Public Health, Oregon Health & Science University, Portland, OR 97239, USA

[5]U.S. Army ERDC-CRREL, Hanover, NH, USA

[*]Corresponding author:

Dr. Wenqing Xu

Email: wenqing.xu@villanova.edu

Phone: 610-519-8549

Supporting Information File for Environmental Science & Technology

Contents: 11 pages, 5 Tables, 13 Figures

Text S1. Analytical methods.

A Waters Acquity H-Class UPLC was used with an Acquity UPLC BEH C18 column (2.1 mm × 50 mm, 1.7 μm) maintained at 35°C. Sample injection volume was 5.0 μL; separations were achieved using a gradient elution (**Table S3**). The total flow rate was 0.40 mL·min^{-1}. A Waters Xevo G2-S qTOF-MS equipped with an electrospray ionization (ESI) source was operated in negative polarity and resolution mode with the following parameters: capillary voltage = 2.50 kV; sampling cone = 40 V; source offset = 80 V; source T = 120°C; desolation T = 250°C; cone gas (N_2) = 80 L·h^{-1}; desolation gas (N_2) = 800 L·h^{-1}. MS^e experiments were employed in which exact mass data for parent ions (MS) and daughter ions (MS/MS; collision energy ramped from 15 to 30 V) were collected concurrently for mass-to-charge ratios ranging from 100 – 600 Da.

Table S1. Elemental analysis of different carbon types.

	[d] Elemental composition (percentage by weight)					Atomic Ratio			[e] Surface Area ($m^2 \cdot g^{-1}$)
**Carbon**	**C**	**O**	**H**	**N**	**Ash**	**O/C**	**H/C**	**N/C**	
**Almond Shell Char**	64.98	12.42	2.38	0.65	13.85	0.19	0.04	0.01	8.8
[a] **PAC**	77.03	8.08	1.37	0.78	12.74	0.10	0.02	0.10	1700
[b] **PAC-QA$_{Phys}$**	75.55	13.11	2.35	1.66	N.A.	0.17	0.03	0.02	1146.7
[c] **PAC-QA$_{Chem}$**	73.09	11.83	1.61	0.76	8.66	0.16	0.02	0.01	62.2
**Graphite Powder**	100.2	<1.0	<1.0	<1.0	N.A.				11.9

[a] PAC = Powdered activated carbon
[b] PAC-QA$_{Phys}$ = Physically modified activated carbon
[c] PAC-QA$_{Chem}$ = Chemically modified activated carbon
[d] Elemental analysis performed by Galbraith Laboratories (Knoxville, TN)
[e] Surface area performed by New Jersey Institute of Technology (Newark, NJ)

Table S2. Extraction efficiencies (%) of DNAN on different carbon types.

**Carbon Type**	**Extraction Efficiency (%)**		
	**24 hours**	**48 hours**	**Average**
**Graphite**	82.0±0.6	82.1±0.1	82.1±0.1
**AS char**	54.4±1.6	58.0±0.1	56.2±0.1
**PAC**	24.0±0.6	18.5±0.7	21.3±3.4
**PAC-QA$_{Phys}$**	31.7±0.6	30.4±0.1	31.1±1.2
**PAC-QA$_{Chem}$**	91.8±3.1	90.9±0.7	91.3±0.3

Table S3. UPLC Mobile Phase Gradient used with the UPLC-qTOF-MS analyses.[a]

**Time (min)**	**Component A (%)**	**Component B (%)**
**0**	95	5
**1**	95	5
**12**	5	95
**13**	95	5
**14**	95	5

[a] Component A contained 18 MΩ•cm water (95 vol%), LC-MS grade methanol (5 vol%), and 2 mM ammonium acetate. Component B contained LC-MS grade methanol and 2 mM ammonium acetate. Total mobile phase flow rate was maintained at 0.40 mL•min^{-1}.

Table S4. Observed reaction rate constants (k_{obs}) and half-life ($t_{1/2}$) for DNAN hydrolysis with and without 22 g·L^{-1} graphite at 25°C,45°C and 65°C for 21 days at pH 11.5.

**Temperature (°C)**	$\boldsymbol{k_{obs,w/o\ graphite}}$ **(d^{-1})**	$\boldsymbol{k_{obs,w\ graphite}}$ **(d^{-1})**	$\boldsymbol{t_{1/2,wo\ graphite}}$ **(d)**	$\boldsymbol{t_{1/2,w\ graphite}}$ **(d)**
25	0.004 ± 0.001	0.021 ± 0.001	184.8 ± 46.2	33.1 ± 1.6
45	0.022 ± 0.002	0.035 ± 0.001	25.8 ± 1.9	18.3 ± 0.5
65	0.041 ± 0.002	0.073 ± 0.004	16.95 ± 0.9	9.5 ± 0.5

Table S5. Observed reaction rate constants (k_{obs}) and half-life ($t_{1/2}$) for DNAN hydrolysis with 10 g·L^{-1} of different carbon powders at pH 11.5 at 25°C.

**Sample name**	$\boldsymbol{k_{obs}}$ **(d^{-1})**	$\boldsymbol{t_{1/2}}$ **(d)**
**No PCM**	0.004 ± 0.001	184.8 ± 46.2
**Graphite**	0.01±0.001	73.8±7.8
**AS char**	0.02±0.001	37.8±2.1
**PAC**	0.04±0.01	16.3±1.9
**PAC-QA$_{Phys}$**	0.06±0.01	12.7±1.2
**PAC-QA$_{Chem}$**	0.28±0.03	2.5±0.3

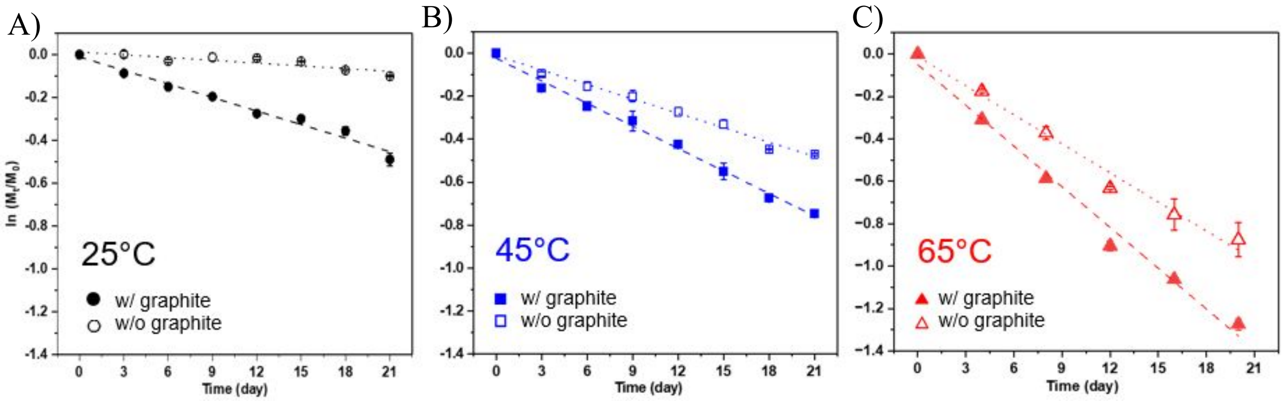

Figure S1. DNAN degradation in the presence and absence of 22 g·L^{-1} graphite for 21 days at pH 11.5 at A) 25°C (●,○), B) 45°C (■,□), and C) 65°C (▲,△). Initial concentration of DNAN was 50 µM. The reported data were derived from duplicate samples based on the standard error of the regression to determine a 95% confidence interval.

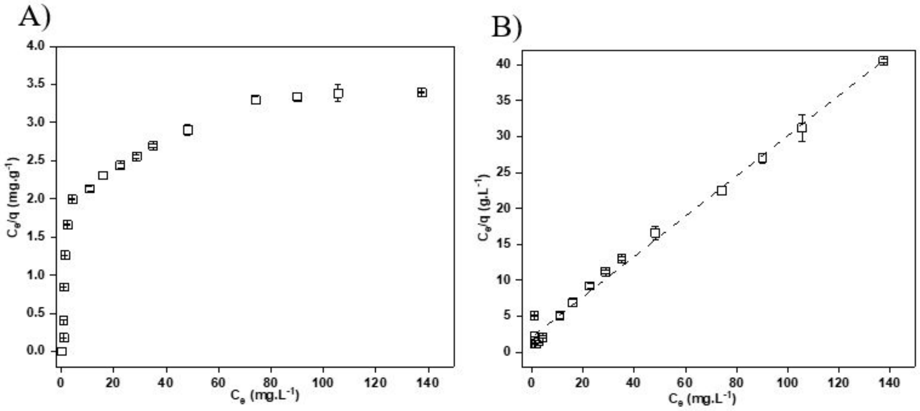

Figure S2: A) Adsorption isotherm generated for DNAN in the presence of 22 g·L^{-1} graphite powder in pH 3 adjusted deionized water after a 2-day equilibration period. The adsorption isotherm for DNAN in the presence of 22 g·L^{-1} graphite powder linearly fit to the Langmuir model. The reported data were derived from duplicate samples.

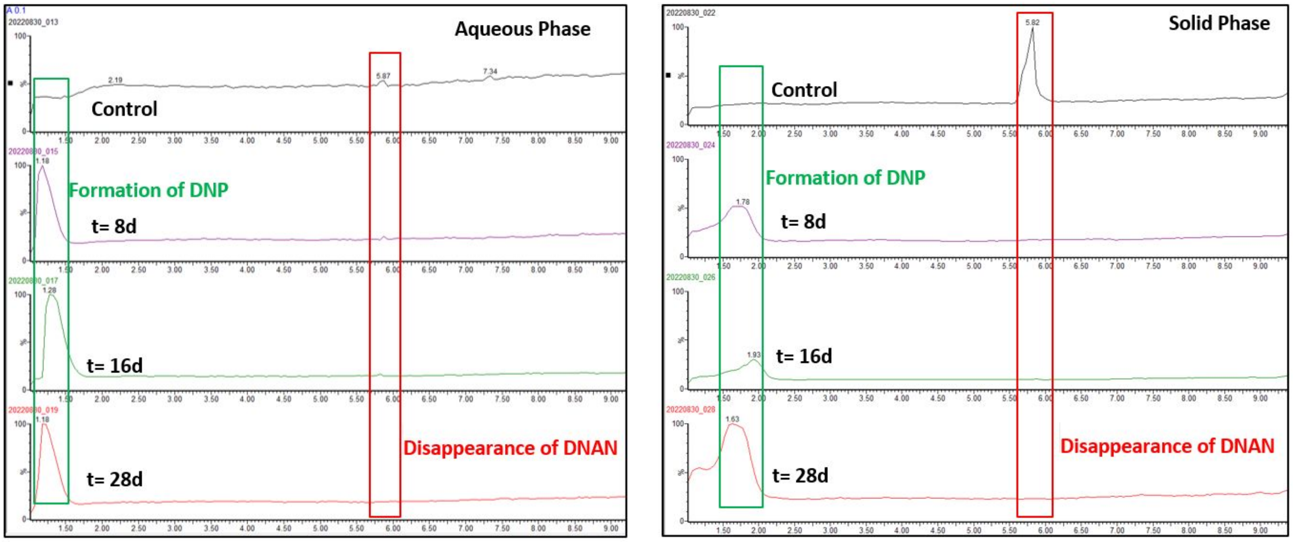

Figure S3. Formation of DNP in the aqueous phase and solid phase during the degradation of DNAN in the presence of graphite powder. Reaction conditions: $[DNAN]_0$ = 50 µM, [graphite powder] = 22 g·L^{-1}, T = 65ºC, pH = 11.5 (20 mM phosphate-carbonate buffer).

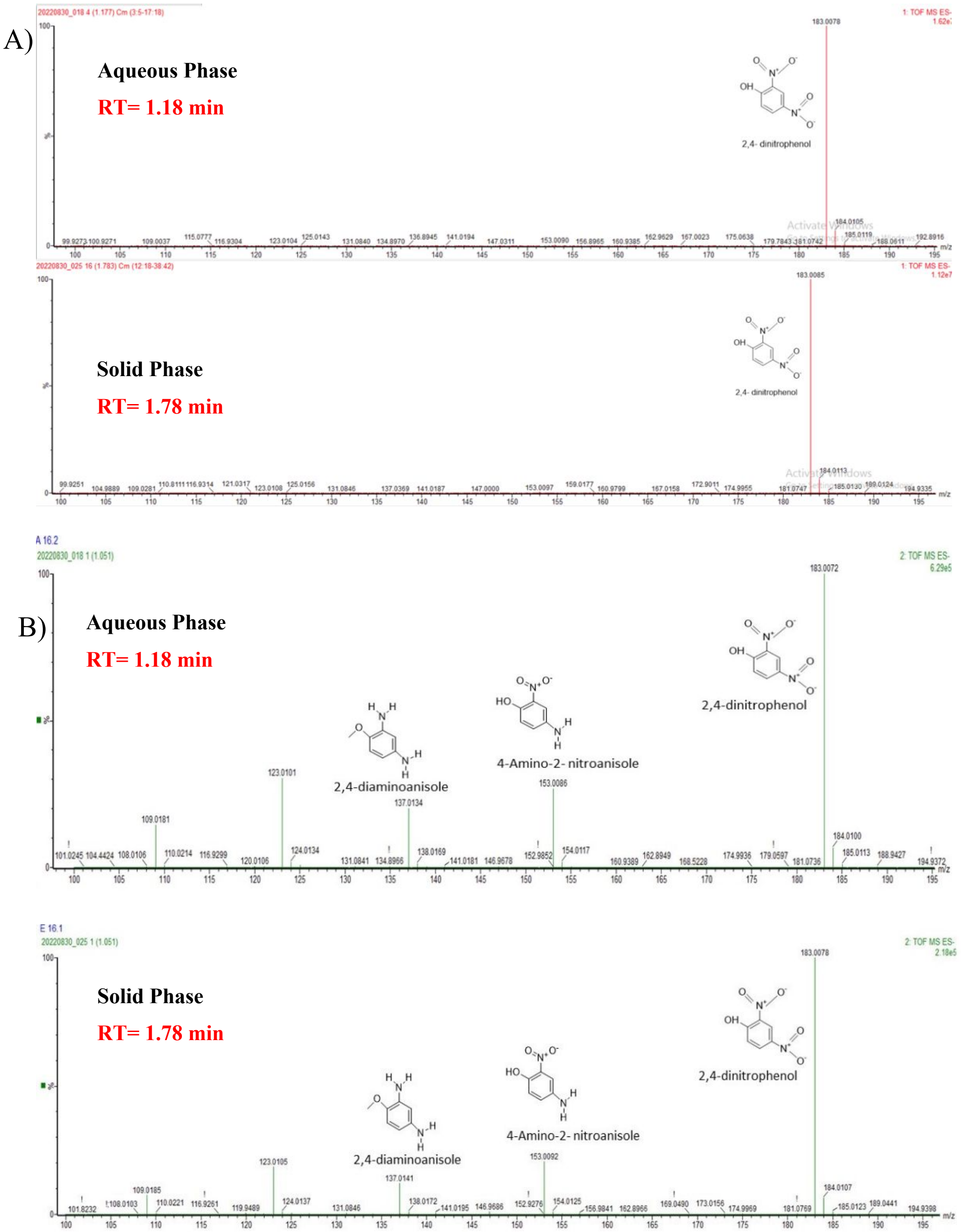

Figure S4. MS (parent ions) and MS/MS (daughter ions) images from UPLC-ESI(+)-qTOF-MS experiments A) with no collision energy and B) with collision energy (15 to 30 V) for possible transformation products. Reaction conditions: $[DNAN]_0$ = 50 µM, [graphite powder] = 22 g·L^{-1}, T = 65ºC, pH = 11.5 (20 mM phosphate-carbonate buffer).

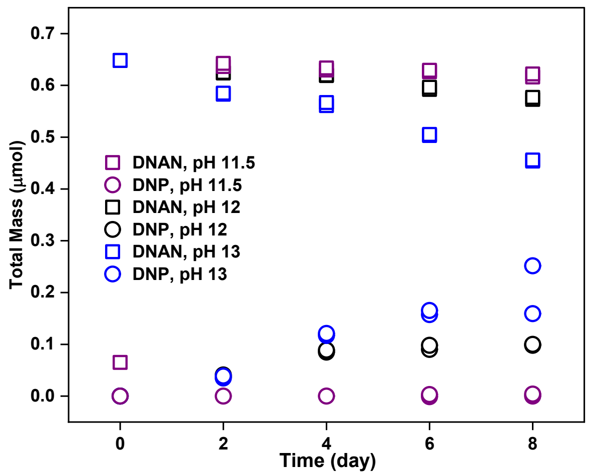

Figure S5. The degradation of DNAN and formation of DNP in the homogeneous solution at pH 11.5, 12, and 13 in 20 mM phosphate-carbonate buffer at 25°C over 8 days. The initial mass of DNAN was 0.65 µmol. The reported data were derived from duplicate samples.

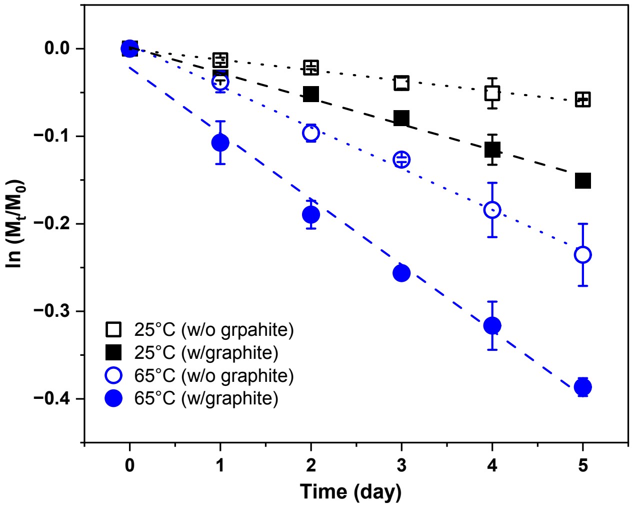

Figure S6. DNAN degradation in the presence and absence of 22 g·L^{-1} graphite over 5 days at 25 and 65°C and at pH 11.5 in 20 mM phosphate buffer. The initial concentration of DNAN was 50 µM. The reported data were derived from duplicate samples based on the standard error of the regression to determine a 95% confidence interval.

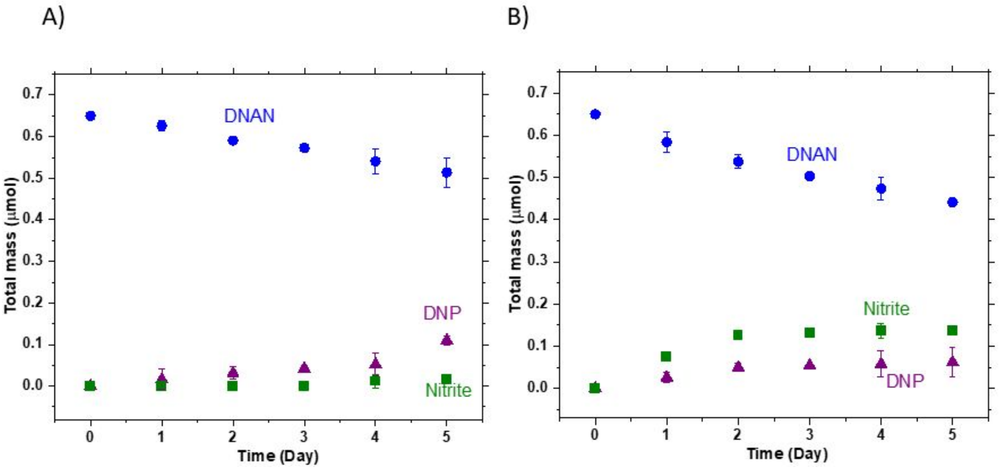

Figure S7. The decay of DNAN and formation of nitrite and DNP in the A) absence and B) presence of 22 g·L^{-1} crystalline graphite powder at 65°C over 5 days at pH 11.5 in 20 mM phosphate buffer. The initial concentration of DNAN was 50 µM. The reported data were derived from duplicate samples.

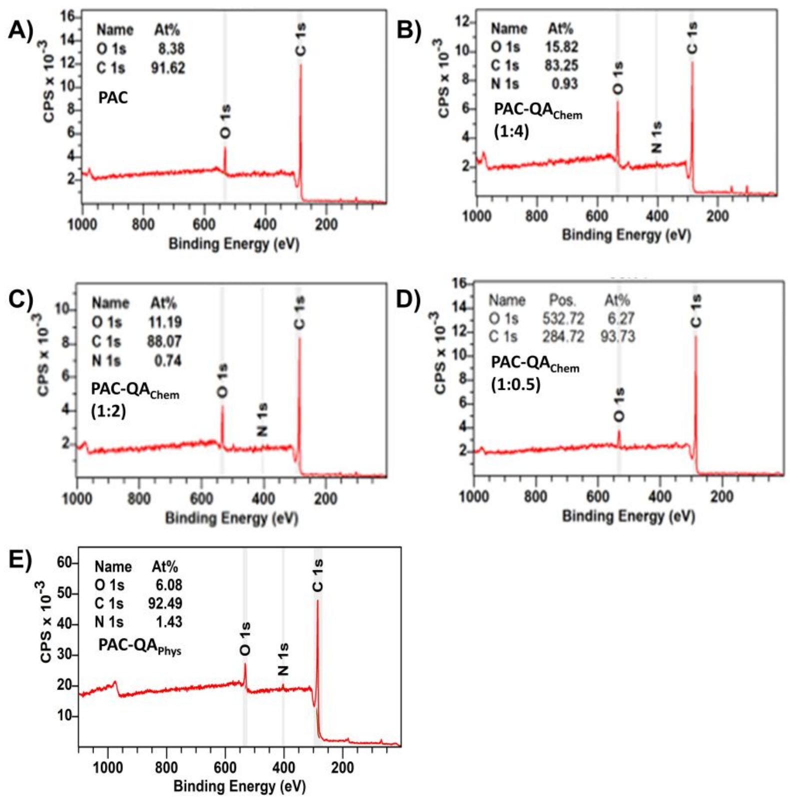

Figure S8. XPS survey spectra of A) PAC alone, PAC-QA$_{Chem}$ prepared using three different ratios (by weight) of PAC-OH and GTAC B) 1:4, C) 1:2, D) 1:0.5 and E) PAC-QA$_{Phys}$.

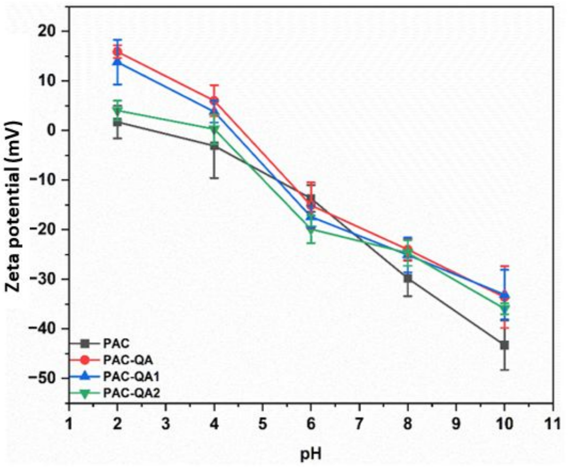

Figure S9. Zeta potential for PAC-QA_{Chem} with reagent ratio of 1:4 (PAC-QA), 1:2 (PAC-QA1) and 1:0.5 (PAC-QA2) (by weight) as a function of pH. The reported data were derived from duplicate samples.

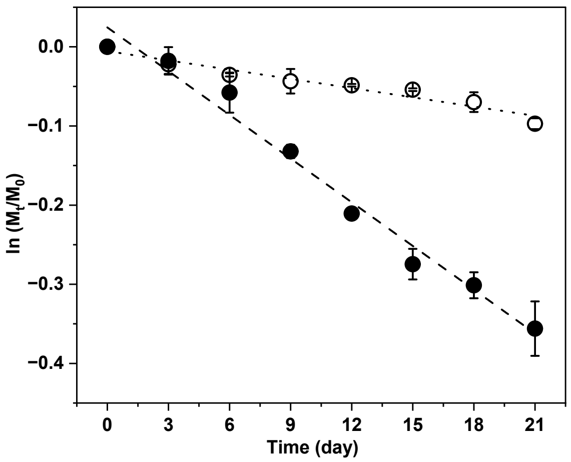

Figure S10. DNAN degradation in the presence (●) and absence (○) of 10 g·L^{-1} almond shell char at pH 11.5 over 21 days at 25°C. Initial concentration of DNAN was 50 µM. The reported data were derived from duplicate samples based on the standard error of the regression to determine a 95% confidence interval.

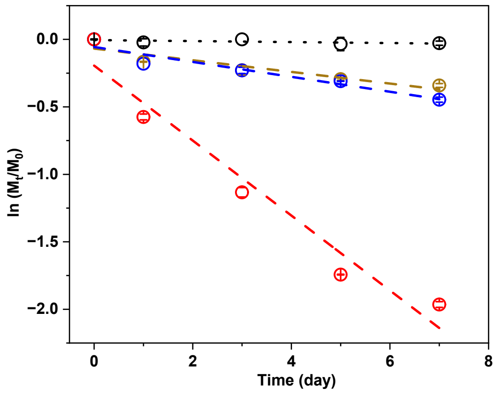

Figure S11. DNAN degradation in the absence (○) and presence of 10 g·L^{-1} PAC (○), PAC-QA$_{Phys}$ (○), or PAC-QA$_{Chem}$ (○) for 7 days at pH 11.5 and 25°C. Initial concentration of DNAN was 50 µM. The reported data were derived from duplicate samples based on the standard error of the regression to determine a 95% confidence interval.

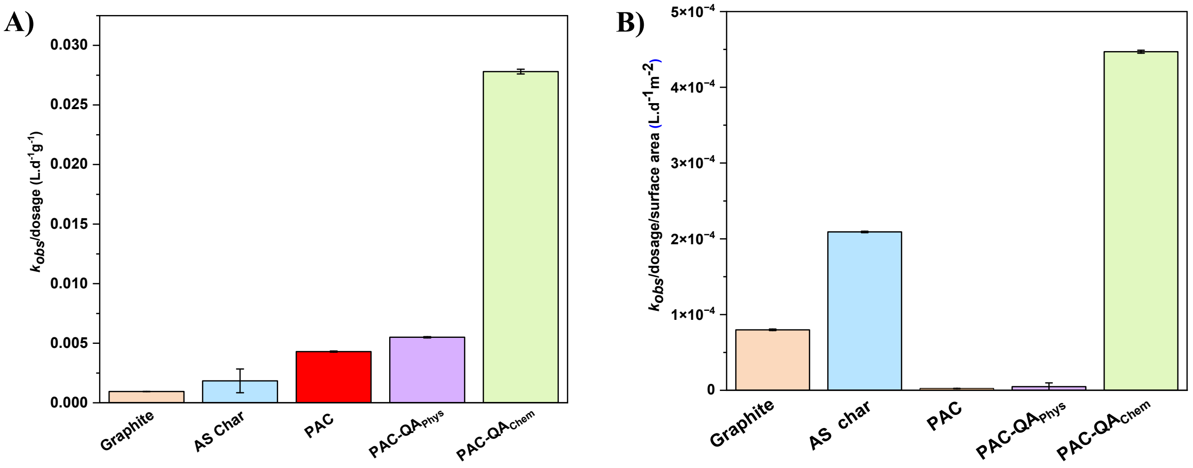

Figure S12. Pseudo first-order DNAN decay kinetics with graphite, AS char, PAC, PAC-QA$_{Phys}$ and PAC-QA$_{Chem}$ normalized to A) PCM dosage, and B) PCM dosage and surface area. Reaction conditions: [DNAN]$_0$ = 50 µM, solid to liquid ratio = 10 g·L^{-1}, T= 25°C , pH= 11.5 (20 mM phosphate-carbonate buffer). The reported data were derived from duplicate samples.

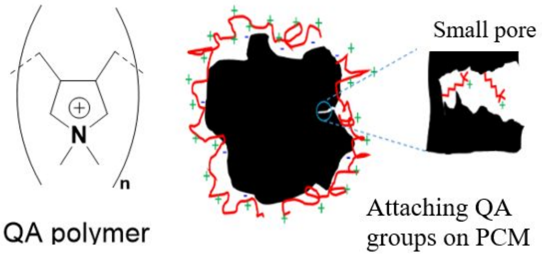

Figure S13. PCM modification with quaternary ammonium (QA) groups.
